# Comparative Functional Morphology of Human and Chimpanzee Feet Based on Three-Dimensional Finite Element Analysis

**DOI:** 10.3389/fbioe.2021.760486

**Published:** 2022-01-13

**Authors:** Kohta Ito, Tomoya Nakamura, Ryo Suzuki, Takuo Negishi, Motoharu Oishi, Takeo Nagura, Masahiro Jinzaki, Naomichi Ogihara

**Affiliations:** ^1^ Department of Mechanical Engineering, Faculty of Science and Technology, Keio University, Yokohama, Japan; ^2^ Graduate School of Human Sciences, Osaka University, Suita, Japan; ^3^ Department of Biological Science, Graduate School of Science, The University of Tokyo, Tokyo, Japan; ^4^ Department of Veterinary Medicine, Azabu University, Sagamihara, Japan; ^5^ Department of Clinical Biomechanics, Keio University School of Medicine, Tokyo, Japan; ^6^ Department of Radiology, Keio University School of Medicine, Tokyo, Japan

**Keywords:** foot biomechanics, evolution, bipedal locomotion, vertical free moment, foot musculoskeletal model

## Abstract

To comparatively investigate the morphological adaptation of the human foot for achieving robust and efficient bipedal locomotion, we develop three-dimensional finite element models of the human and chimpanzee feet. Foot bones and the outer surface of the foot are extracted from computer tomography images and meshed with tetrahedral elements. The ligaments and plantar fascia are represented by tension-only spring elements. The contacts between the bones and between the foot and ground are solved using frictionless and Coulomb friction contact algorithms, respectively. Physiologically realistic loading conditions of the feet during quiet bipedal standing are simulated. Our results indicate that the center of pressure (COP) is located more anteriorly in the human foot than in the chimpanzee foot, indicating a larger stability margin in bipedal posture in humans. Furthermore, the vertical free moment generated by the coupling motion of the calcaneus and tibia during axial loading is larger in the human foot, which can facilitate the compensation of the net yaw moment of the body around the COP during bipedal locomotion. Furthermore, the human foot can store elastic energy more effectively during axial loading for the effective generation of propulsive force in the late stance phase. This computational framework for a comparative investigation of the causal relationship among the morphology, kinematics, and kinetics of the foot may provide a better understanding regarding the functional significance of the morphological features of the human foot.

## Introduction

To adapt to habitual bipedal locomotion, the human foot has evolved significantly in the course of human evolution (Morton, 1922; Weidenreich, 1923; Morton, 1924; Susman, 1983). The foot of non-human primates, such as chimpanzees, has an opposable hallux that faces the other four digits to allow the grasping of objects. By contrast, the hallux of the human foot is aligned in parallel with the other four digits; as such, the prehensile function is not afforded. In addition, the human foot exhibits unique morphological features, such as a longitudinal arch with an enlarged, robust calcaneus, and a well-developed plantar aponeurosis, which allows mechanical energy to be stored in the form of elastic energy and successively released during the contact of each foot (Ker et al., 1987; Bramble and Lieberman, 2004; Stearne et al., 2016). Additionally, a curved transverse arch, which can increase the stiffness of the human foot, has been suggested recently (Venkadesan et al., 2020).

During the stance phase of gait, the feet are the only parts of the body that mechanically interact and are in direct contact with the ground. Because human bipedal locomotion is a mechanical phenomenon that translates the body center-of-mass while avoiding a fall by the appropriate control of reaction forces applied to the body from the ground, the unique morphological specialization of the human foot is expected to be closely associated with the acquisition of habitual bipedal locomotion during the course of human evolution. The fact that the human foot is morphologically distinct from that of other primates indicates that the modification of the foot structure is vital to the evolution of human bipedal locomotion.

To understand the relationship between foot morphology and bipedal locomotion, the kinematics and kinetics of the foot during locomotion must be elucidated. Therefore, many studies have been conducted to measure the kinematics and kinetics of the foot bones during human walking (e.g., Nester, 2009; Deschamps et al., 2011, 2017; Kessler et al., 2019; Leardini et al., 2019). However, experimental analysis of form–function relationships of the foot bones is not methodologically trivial because of the complexity of the foot skeletal system; this applies similarly to the difficulty associated with the measurement of the kinematics and kinetics of foot bones during locomotion, since any intervention to measurements, such as the use of bone pins and X-ray fluoroscopy, might be invasive and perturb the normal behaviors of the foot during locomotion. However, computer simulations of the entire human foot based on physiologically realistic finite element (FE) models and loading conditions can clarify the form–function relationship of the human foot. Such simulations may enable the evaluation and prediction of changes in the foot function resulting from the virtual alteration of the foot morphology, thereby facilitating investigations into the effects of differences in the foot morphology to the mechanical performance of the foot. Therefore, a numerical model of the human foot using an FE method has been developed recently to simulate the basic biomechanics of the foot (Chen et al., 2001; Cheung and Zhang, 2005; Cheung and Zhang, 2008; Gu et al., 2010a; Gu et al., 2010b; Chen et al., 2012; Luximon et al., 2012; Akrami et al., 2018). More recently, dynamic FE analyses of the foot during walking were performed to clarify the dynamic and functional interaction of the foot complex with the ground during walking (Qian et al., 2013) and running (Chen et al., 2019). However, to the best of our knowledge, an FE model of the non-human primate foot for a better understanding of the functional morphology and evolution of the human foot has not been developed.

The aim of this study was to develop three-dimensional (3D) FE models of the entire foot of humans and chimpanzees to comparatively investigate the morphological adaptation of the human foot. Specifically, the axial loading of the foot without any tendon traction was simulated to clarify the innate mobility and mechanical properties of the foot. In addition, physiologically realistic loading conditions of the foot during quiet standing were simulated, and the difference in load transmission through the foot bones of humans and chimpanzees was investigated. Particularly, we computationally tested the hypothesis if there are any differences in foot bone movements, force distributions, vertical free moments, and force-displacement curves between the human and chimpanzee foot during axial loading and simulated quiet standing. Dynamic simulations of human and chimpanzee walking based on the developed foot models will be performed in a successive study.

## Methods

### Human Foot Model

**FIGURE 1:**
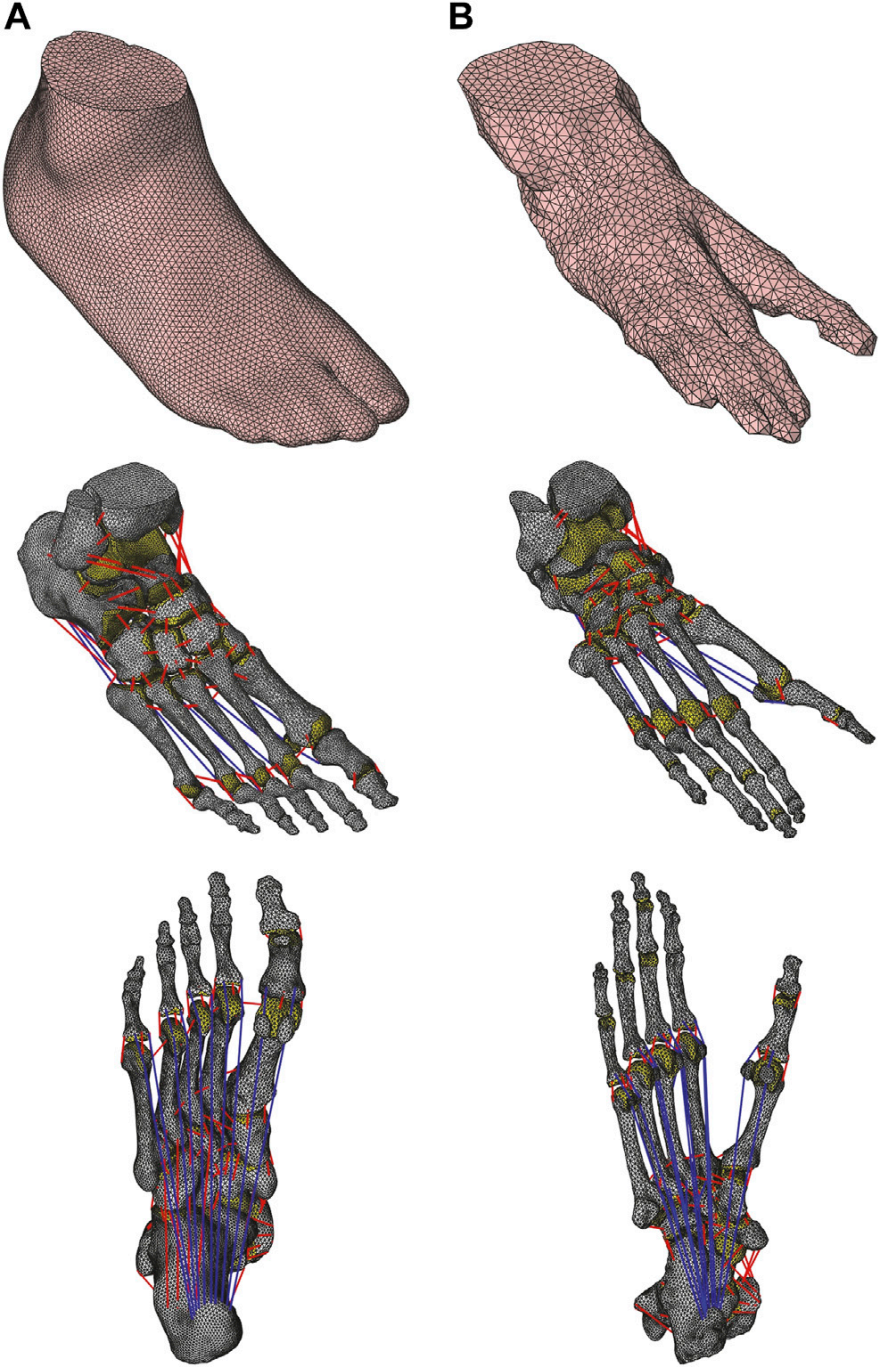
Finite element models of human **(A)** and chimpanzee foot **(B)** with and without soft tissue. Plantar view of foot models show plantar aponeurosis. Cartilages are illustrated as yellow areas; ligaments and plantar aponeurosis are indicated by red and blue lines, respectively.

A 3D FE model of the human foot was developed based on CT scan data of a healthy male participant (age, 42 years; height, 172 cm; weight, 72 kg; foot length, 25.5 cm) with no history of orthopedic or neuromuscular impairments. The CT scan was performed in the supine position without any weight bearing. Cross-sectional images were reconstructed at 0.25 mm intervals, with a pixel size of 0.507 mm. Subsequently, the serial images were transferred to the Analyze 9.0 software (Biomedical Imaging Resource, Mayo Clinic, Rochester, MN, United States) to generate 3D models of the external surfaces of the foot bones and the entire foot surface (Figure 1). Some adjacent phalanges were extracted as one entity because the spatial resolution of the CT data was not sufficiently small to separate the bones. Three hallux sesamoids (two at the metatarsophalangeal joint and one at the interphalangeal joint) were extracted and included in the model. Therefore, there were a total of 23 bones constituting the human foot model: tibia, fibula, talus, calcaneus, navicular, cuboid, three cuneiforms, five metatarsals, proximal and distal hallucal phalanges, four fused phalanges of the second to fifth rays, and three sesamoids. Subsequently, the surface models, which were in the stl format, were converted to iges files using Rhinoceros 3D (Robert McNeel & Associates, Seattle, WA, United States). The volume enclosed by each surface was meshed with four-node tetrahedral elements using HyperMesh 2017 (Altair Engineering, Troy, MI, United States). The encapsulated soft tissues between the outer foot surface and bone surface were meshed with tetrahedral elements. To simulate the articular surface-to-surface contact behaviors, elements corresponding to the articular cavity were manually removed, and the articular surface-to-surface was simulated using a frictionless contact model. In several previous studies (e.g., Guo et al., 2009), unphysiological contact models that do not allow the separation or relative motion of bones on the contact surface were applied; however, such unphysiological contact models were not used in the present study. The horizontal floor was modeled as a rigid wall, and the contact between the plantar surface of the foot and floor was modeled using a contact model with friction, with the coefficient of static and dynamic friction set to 0.6 (Chen et al., 2010).

The bones were represented as an isotropic linear elastic material, and the Young’s modulus, Poisson’s ratio, and density were set to 7,300 MPa, 0.3, and 0.0015 g/mm^3^, respectively, based on previous studies (Chen et al., 2010; Qian et al., 2013). The cartilage is often modeled as a thin cartilaginous layer of 1.0–1.5 mm thickness on the articular surfaces (Shepherd and Seedhom, 1999), but here the bone elements corresponding to articular surfaces were manually selected and assigned as articular cartilage, and the Young’s modulus, Poisson’s ratio, and density were set to 10 MPa, 0.4, and 0.002 g/mm^3^, respectively, based on previous studies (Gefen, 2003; Qian et al., 2013). The encapsulated soft tissue was defined as a hyperelastic Ogden material, whose strain energy potential function *U* can be expressed as

$$U=\frac{2C}{\alpha^{2}}\left(\lambda_{1}^{\alpha }+\lambda_{2}^{\alpha }+\lambda_{3}^{\alpha }-3\right),$$ (1)

where λ is the deviatoric principal stretch; *C* and *α* are coefficients, whose values were determined to be 0.0102 MPa and 8.04, respectively, based on *in vivo* parameter identification using a spherical indentation and an analytical contact mechanics model (Suzuki et al., 2017). The density was determined to be 0.937 × 10^−3^ g/mm^3^ (Qian et al., 2013). The material was assumed to be incompressible (i.e., Poisson’s ratio = 0.5). However, because it was numerically difficult to impose this value, a Poisson’s ratio of 0.475 was used.

The outer surface of the foot encapsulating the soft tissue was meshed with three-node triangular shell elements to represent the skin. The thickness of the skin was modelled as a uniform layer with a thickness of 1 mm, based on published values for the planta pedis (Strzalkowski et al., 2015). The material properties of the skin were represented as a hyperelastic Ogden material, and coefficients *C* and *α* were determined to be 0.122 MPa and 18, respectively (Gu et al., 2010a). The density and Poisson’s ratio of the skin were assumed to be identical to those of the soft tissue.

A total of 95 ligament elements were attached to the model (Supplementary Material). Ligaments were represented as tension-only spring elements connecting the origin and insertion points estimated from the anatomical atlas (Schuenke et al., 2006; Drake et al., 2008). The tensile force generated by the *i*th ligament *F*_*i*_ can be calculated as follows:

$$F_{i}=\frac{EA_{i}}{{\bar{L}}_{i}}\max\left(L_{i}-{\bar{L}}_{i},0\right),$$ (2)

where *E* is the Young’s modulus of the ligament; $A_{i}$ , $L_{i}$ , and ${\bar{L}}_{i}$ are the cross-sectional area, length, and natural length of the *i*th ligament, respectively. The natural length was estimated to be 1.05 times longer than the length of the ligament when the foot was in the CT-scanned posture, as the foot was slightly plantarflexed owing to gravity because the foot was scanned in the supine position. The Young’s modulus and density of the ligaments were determined to be 260 MPa and 0.001 g/mm^3^, respectively (Chen et al., 2010; Chen et al., 2019). The cross-sectional areas of the ligaments in the foot were determined as described in Supplementary Material based on the method by Mkandawire et al. (2005). However, the cross-sectional areas of the ligaments reported by Mkandawire et al. (2005) were much larger than those reported in the frequently cited paper by Siegler et al. (1988). Therefore, we multiplied the cross-sectional areas by a scaling factor of 0.576, which was reported by Mkandawire et al. (2005). The scaling factor was calculated based on the ratio of posterior tibiotalar ligament cross-sectional areas obtained in the abovementioned two studies. The cross-sectional areas of ligaments that were not listed in the paper by Mkandawire et al. (2005) were determined by referring to those of neighboring ligaments. If a ligament is represented by multiple elements, then the cross-sectional area is divided accordingly. The cross-sectional areas of the plantar plate and collateral ligament were determined to be 30 (Maas et al., 2016) and 3 mm^2^, respectively, assuming that the cross-sectional area of the latter is one-tenth that of the former. The cross-sectional areas of the ligaments were typically assumed to be identical in previous studies (e.g., Cheung et al., 2005; Chen et al., 2010); however, we incorporated the size difference of the ligaments in our current study.

We represented the plantar aponeurosis (PA) using 10 tension-only spring elements connecting the origin and insertion points via the sesamoids in the hallux, as shown in Figure 1. To avoid the penetration of the second to fifth PA elements during the dorsiflexion of the MP joints, we defined two intermediate points on the plantar articular surface of each metatarsal head. Therefore, each PA element comprised distal (phalangeal) and proximal (plantar) elements. The tensile forces generated by the *j*th proximal and distal PA elements, $F_{j}^{P}$ and $F_{j}^{D}$ , respectively, can be calculated as follows:

$$F_{j}^{P}=K_{j}^{P}\max\left(L_{j}^{P}-{\bar{L}}_{j}^{P},0\right)$$ (3)

$$F_{j}^{D}=K_{j}^{D}\max\left(L_{j}^{D}-{\bar{L}}_{j}^{D},0\right)$$ (4)

where $K_{j}^{P}$ and $K_{j}^{D}$ , $L_{j}^{P}$ and $L_{j}^{D}$ , and ${\bar{L}}_{j}^{P}$ and ${\bar{L}}_{j}^{D}$ are the spring constant, length, and natural length of the *j*th proximal and distal PA element, respectively. The length of the proximal PA elements when the foot was in the CT-scanned posture was assumed to be the natural length of the PA. The natural length of the distal element was estimated to be 0.75 times shorter than that in the CT-scanned posture, since the metatarsophalangeal joints were slightly dorsiflexed when the foot was CT scanned. The spring constant of the proximal element was estimated based on Caravaggi et al. (2009), who reported that the tensile force of the PA was approximately 1.5 times the body weight when the strain was 0.05. Assuming a body weight of 70 kg, the spring constant of each proximal element $K_{j}^{P}$ was calculated as follows:

$$K_{j}^{P}=\frac{\left(70\right)\left(9.8\right)\left(1.5\right)}{\left(10\right)\left(0.05\right){\bar{L}}_{j}^{P}}$$ (5)

The natural length of the distal element was much shorter than that of the corresponding proximal element. Therefore, the spring constant of the *j*th distal element $K_{j}^{D}$ equivalent to the corresponding proximal element can be calculated as follows:

$$K_{j}^{D}=K_{j}^{P}\times \left({\bar{L}}_{j}^{P}/{\bar{L}}_{j}^{D}\right)$$ (6)

The line density of each PA element was calculated based on the cross-sectional area [29.07 mm^2^, one-tenth of the cross-sectional area of the PA reported by Chen et al. (2010)] and the density of each element (0.001 g/mm^3^) (Chen et al., 2019).

### Chimpanzee Foot Model

A 3D FE model of the chimpanzee foot was developed based on the CT scan data of a frozen cadaveric lower leg (female, age 44 years at the time of death; weight not available; foot length, 25.2 cm) (Figure 1). Cross-sectional images were reconstructed at 0.25 mm intervals, with a pixel size of 0.25 mm. The chimpanzee model was developed in the same manner as described for the human foot model. However, the following differences existed between the human and chimpanzee foot models: 1) The chimpanzee foot did not exhibit an anterior talofibular ligament and a transverse metatarsal ligament between the first and second metatarsals (Raven, 1936; Lovejoy et al., 2009); 2) the long plantar ligament of the chimpanzee foot originated from the plantar surface of the cuboid, whereas that of the human foot originated from the anteroplantar surface of the calcaneal tuberosity (Gomberg, 1985); and 3) the chimpanzee foot had an additional sesamoid for each second, third, and fourth digit, as well as two additional sesamoids for the fifth digit; therefore, all PA elements were modeled with the sesamoids. The PA of the chimpanzee foot was previously suggested to be rudimentary (Lovejoy et al., 2009), but a recent study estimated the stiffness of the PA of chimpanzees to be about one-half of that of humans (Wareing, 2016). Therefore, the spring constants of the PA were assumed to be one-tenth (10%) and one-half (50%) of those of the human values in the chimpanzee foot. The length of the ligaments and the PA elements when the foot was in the CT-scanned posture was assumed to be the natural length of the ligaments and PA elements, as the chimpanzee cadaver foot was scannable via CT in a natural plantigrade posture. Again, some adjacent phalanges were extracted as one entity. Therefore, there were a total of 31 bones constituting the chimpanzee foot model: tibia, fibula, talus, calcaneus, navicular, cuboid, three cuneiforms, five metatarsals, proximal and distal hallucal phalanges, one, three, two and two phalangeal bones of the second, third, fourth, and fifth rays (proximal, middle and distal phalanges were fused in the second ray and the middle and distal phalanges were fused in the fourth and fifth rays), and seven sesamoids.

The female chimpanzee foot is much smaller than the human foot. To facilitate biomechanical comparisons, the chimpanzee foot was enlarged by a factor of 1.323, i.e., the cube root of the total bone volume of the human foot divided by that of the chimpanzee foot. The material parameters were assumed to be identical for both the human and chimpanzee models.

### FE Analysis

In the present study, the mechanical interactions of the human and chimpanzee feet with the ground were simulated via an explicit dynamic analysis with discrete energy relaxation. An explicit FE analysis was performed using RADIOSS 2019 (Altair Engineering, Troy, MA, United States). The SGI Rackable C2112-4GP3/C1102-GP8 (Reedbush-U/H/L) at the Information Technology Center, The University of Tokyo, was used in this study.

### Experimental Validation of Model

The human foot model was validated against a cadaveric study of the human foot under axial loading (Negishi et al., 2021) to investigate the innate mobility and mechanical properties prescribed in the anatomy and morphology of the human foot. In the experiment, human feet were axially loaded with no tendon tractions, and the 3D movements of the foot bones and tibia owing to vertical compression were quantified using biplane fluoroscopy [see Figure 1 in Negishi et al. (2021)]. The proximal end of the tibia and fibula was fixed to a shaft using a custom-developed socket fabricated using a rubber-like polymer material, and the shaft passed through a linear guide such that the shaft can only move along and rotate around the vertical axis. We reproduced this experiment virtually using the developed human foot model. Specifically, the proximal ends of the tibia and fibula were fixed to a rectangular solid of rubber material (70 mm × 50 mm × 20 mm; Young’s modulus = 4.0 MPa; Poisson’s ratio = 0.475; density = 0.0012 g/mm^3^), and the rubber rectangular solid was fixed to a vertical axis at the midpoint between the proximal ends of the tibia and fibula. The solid was allowed to move only along and around the vertical axis, as in the cadaver experiment. The posture when only the vertical shaft (3.3 kg) was affixed to the specimen was defined as the zero-loading condition. In the experiment, the foot was initially placed from the heel by slightly elevating the forefoot; subsequently, the entire foot was firmly placed in a fully plantigrade position, so that unnatural shear force was not applied to the plantar surface of the foot when creating the initial zero-loading condition. This process was simulated by applying upward forces at the metatarsal head while axially loading the tibia and fibula. This is important for reproducibility of the results as the way we placed the specimen might have affected the mobility of the foot during axial loading. Next, the foot model was loaded axially from the zero-loading condition up to 588 N (60 kg), and the 3D translations and rotations of the foot bones due to axial loading were calculated for comparison. The computing time of this simulation was approximately 15 h.

### Comparisons of Human and Chimpanzee Foot Models

To comparatively investigate the functional morphology of the human foot, two different loading conditions were simulated. First, the axial loading of the foot without any tendon traction was simulated to clarify the innate mobility and mechanical properties of the foot. An axial force of 350 N was applied from the initial position without tendon traction, and a convergent static solution was obtained. Second, physiologically realistic loading conditions of the foot during quiet standing were simulated, and the difference in load transmission through the foot bones of the human and chimpanzee was investigated. For a body mass of 70 kg, a vertical ground reaction force of approximately 350 N was applied to each foot during balanced standing. Simultaneously, a force was generated by the tricep surae muscle (the Achilles tendon), the magnitude of which was estimated to be approximately 50% of the ground reaction force based on a structural analysis of the foot during quiet standing (Cheung et al., 2005). Therefore, a downward force of 525 N and an upward force of 175 N were applied to the tibia and calcaneal tuberosity, respectively, in the present study. Subsequently, dynamic calculations were conducted as follows: An axial force of 350 N was applied from the initial position without tendon traction, and the convergent static solution was obtained; next, an additional 175 N of axial force was applied to the tibia and an upward force was applied to the calcaneal tuberosity to obtain the final static solution. The computing time of this simulation was approximately 15 h.

The same calculation was conducted for the chimpanzee foot model. Since the loading conditions of the foot were of the human quiet standing, this simulation did not physiologically replicate the chimpanzee foot standing bipedally; chimpanzees stand bipedally with bent hips and knees, so that the loading conditions of the foot might be quite different between the human and chimpanzee feet. Instead, by applying the loading conditions of the human quiet sanding, we investigated what would happen if humans stood bipedally with chimpanzee feet, to comparatively extract possible functional significances of the human foot morphology. The initial position of the foot was defined by solving the static equilibrium of the foot when a downward force of 15 N was applied to the tibia, such that the entire plantar surface of the foot touched the floor. The computing time of this simulation was approximately 13 h.

To validate the human model during quiet standing, the calculated plantar pressure distribution was compared with the plantar pressure distribution during balanced standing measured for the participant whose right foot was CT scanned using a tactile sensor system (BIG-MAT1300, Nitta, Tokyo, Japan). However, no corresponding data were available for the chimpanzee foot.

### Bone Kinematics

To quantify the 3D bone kinematics due to axial loading, a bone-fixed local coordinate system was defined such that the three orthonormal axes (xyz) of the local coordinate system at the zero position were aligned with the three orthonormal axes (XYZ) of the global coordinate system. The X-, Y-, and Z-axes corresponded to the inversion–eversion, plantarflexion–dorsiflexion, and internal–external rotation axes, respectively. The origin of the bone coordinate system was defined as the centroid of the corresponding bone. The change in the position and orientation of the bone due to axial loading was quantified using the y–x–z Euler angles, as in Ito et al. (2017). The Euler angles describing the relative rotations during axial loading were calculated as follows:

$$\begin{array}{l} R={\left[M_{Z}\right]}^{T}M_{L}\\ \;=R_{y}\left(\phi \right)R_{x}\left(\theta \right)R_{z}\left(\psi \right), \end{array}$$ (7)

where **R** is the rotational matrix, whose rotational sequence is y, x, and z; **M**_Z_ and **M**_L_ are the orthonormal matrices of the coordinate frames fixed to the bone when the foot was in the zero position and fully loaded, respectively; **R**_y_, **R**_x_, and **R**_z_ and ϕ , θ , and ψ are the rotational matrices and Euler angles around the y-, x-, and z-axes, respectively, which represent plantarflexion–dorsiflexion, inversion–eversion, and internal–external rotations, respectively.

The bone-to-bone angles of the subtalar, talonavicular, and calcaneocuboid joints were defined as the motion of the distal bone coordinate system with respect to the proximal bone coordinate system. The Euler angles describing the relative rotations of the distal bone with respect to the proximal bone were calculated as follows:

$$\begin{array}{l} R={\left[M_{P}\right]}^{T}M_{D}\\ \;=R_{y}\left(\phi \right)R_{x}\left(\theta \right)R_{z}\left(\psi \right), \end{array}$$ (8)

where **M**_P_ and **M**_D_ are the orthonormal matrices of the coordinate frames fixed to the proximal and distal bones, respectively.

### Center of Pressure and Vertical Free Moment

To evaluate the effect of the difference in foot morphology on the mechanical interaction of the foot with the ground during quiet standing, the COP position and the ground reaction moment around the vertical axis of the floor (vertical free moment; VFM) due to the axial loading of the foot were calculated. The position of the COP ${\left(x_{COP},y_{COP}\right)}^{T}$ can be calculated as follows:

$$\begin{array}{l} x_{COP}=\sum_{i}\left(f_{zi}\cdot x_{i}\right)/\sum_{i}f_{zi}\\ y_{COP}=\sum_{i}\left(f_{zi}\cdot y_{i}\right)/\sum_{i}f_{zi}, \end{array}$$ (9)

where ${\left(f_{xi},f_{yi},f_{zi}\right)}^{T}$ is the force exerting on the *i*th plantar node, and ${\left(x_{i},y_{i}\right)}^{T}$ is the position of the *i*th plantar node. The VFM around the COP can be calculated as follows:

$$VFM=\sum_{i}\left\{f_{yi}\cdot \left(x_{i}-x_{COP}\right)\right\}-\sum_{i}\left\{f_{xi}\cdot \left(y_{i}-y_{COP}\right)\right\}$$ (10)

### Elastic Energy Stored in Foot

To evaluate the capacity to store elastic energy in the foot during axial loading, the foot model was axially loaded with no Achilles tendon traction, and a force–displacement curve was obtained. Specifically, the vertical ground reaction force applied to the plantar surface of the foot was plotted against the downward displacement of the tibia. The plots were approximated via an exponential function *y* = *a x* ^*b*^ + *c* using the least-squares method, and the elastic energy stored in the foot due to axial loading up to 350 N was calculated by integrating the force–displacement curve. The downward displacement of the tibia was zero when the foot was in the zero position. Therefore, the y-intercept (*c*) of the curve was not equal to zero.

## Results

### Validation of Human Foot Model

**FIGURE 2:**
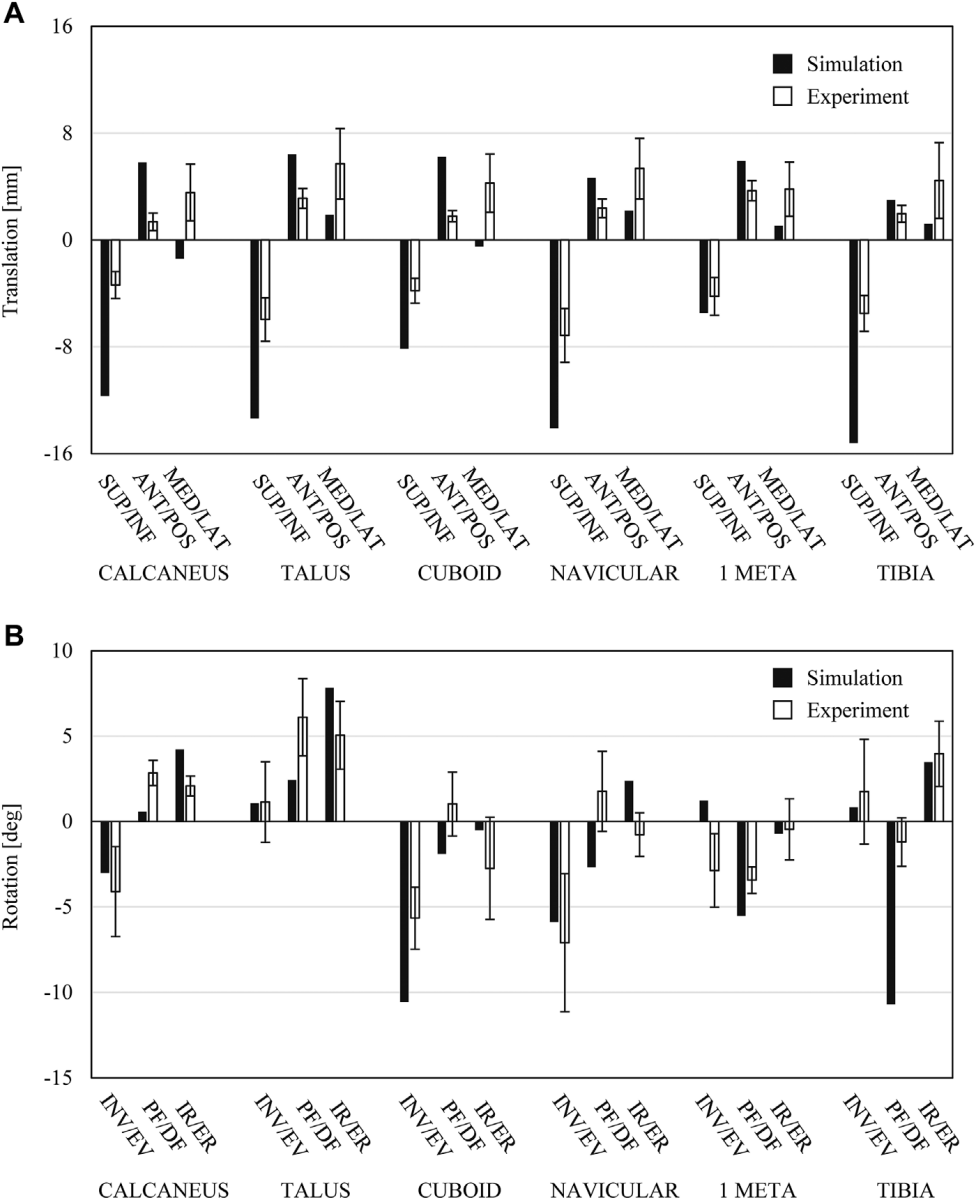
Comparisons between simulated bone movements in human foot model during axial loading and experimentally measured foot bone movements during axial loading (Negishi et al., 2021). **(A)** Translational displacements of foot bones in superoinferior, anteroposterior and mediolateral directions. **(B)** Rotational displacements of foot bones in coronal sagittal and transverse planes.

We first compared the translational and rotational movements of the foot bones during axial loading in the FE simulation with the corresponding experimentally obtained bone movements of cadaver feet (Negishi et al., 2021) to evaluate the simulation model (Figure 2). The magnitudes of the inferior and medial translations of the foot bones were relatively larger and smaller, respectively, in the simulated foot compared with those in the cadaver feet during axial loading. However, the directions of the rotational movements of the foot bones in the simulation were consistent with the measured data. The mean absolute translational differences between the simulated and measured bone movements in the superoinferior, anteroposterior and mediolateral directions were 6.3, 3.0 and 1.8 mm, respectively, and those of the rotational differences in the inversion-eversion, plantar-dorsiflexion, and internal-external rotation were 2.0°, 4.2°, and 1.8°, respectively. Given the fact that the foot model differed from the cadaver specimens used for experimental measurements, the differences were quite small.

**FIGURE 3:**
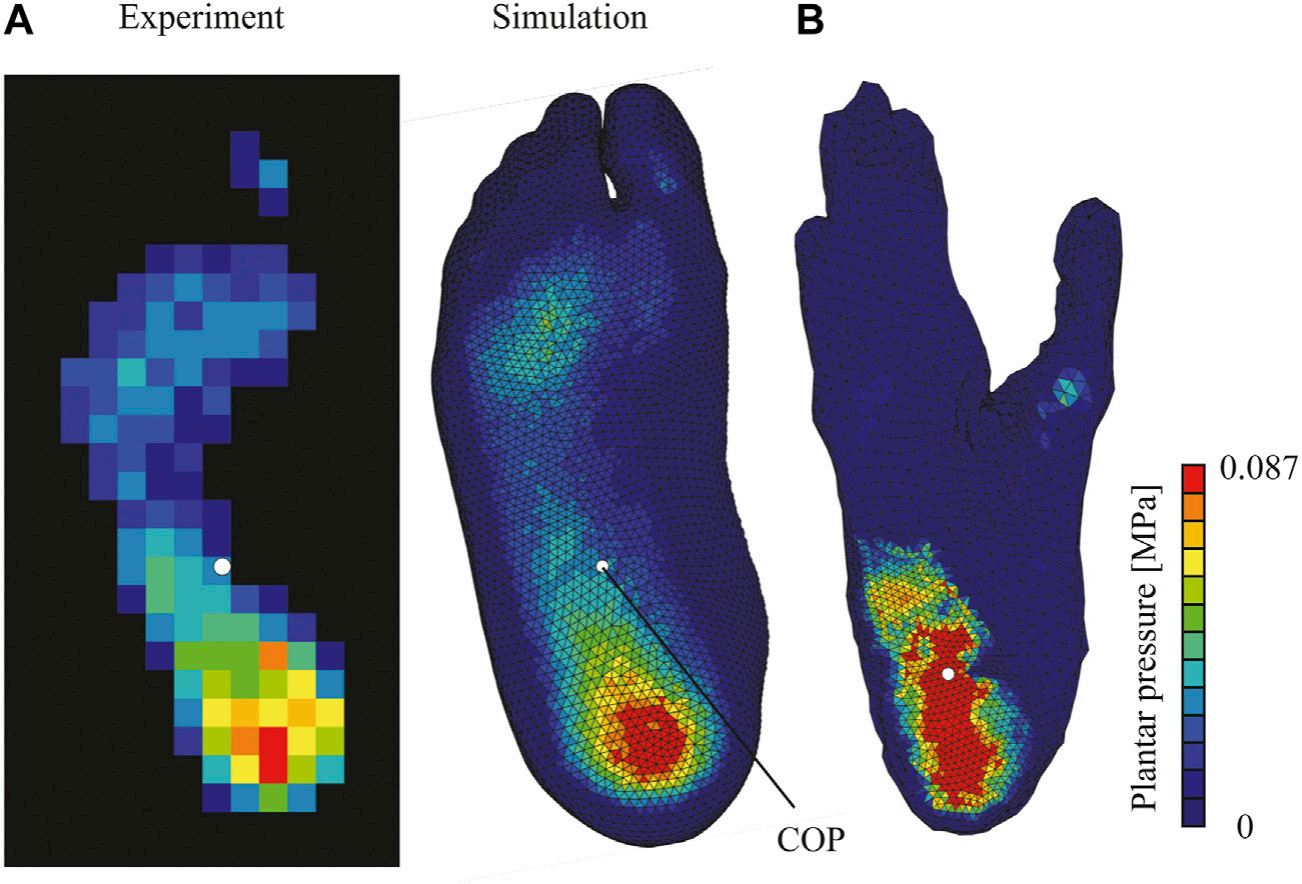
Plantar pressure distributions of human **(A)** and chimpanzee **(B)** feet during simulated quiet standing. Calculated center of pressure (COP) depicted as white circle. Experimentally measured plantar pressure distribution of human during quiet standing presented for comparison.

In addition, the simulated plantar pressure distribution pattern during quiet standing was compared with that measured from an adult male whose right foot was CT scanned and used to develop the FE model. The simulated pressure distribution pattern and the center of pressure were generally concordant, if not matched perfectly, with the measured data (Figure 3A). Hence, our simulation framework successfully reproduced the basic biomechanical features of the human foot with reasonable accuracy.

### Comparisons Between Human and Chimpanzee Feet During Quiet Standing

The calculated results of the chimpanzee foot model with the spring constants of the PA assigned to be one-tenth (10%) and one-half (50%) of the human values are very similar to each other. Therefore, the results of the 10% model were presented here for the comparisons with those of the human model. See Supplementary Material for the results of the 50% model.

**FIGURE 4:**
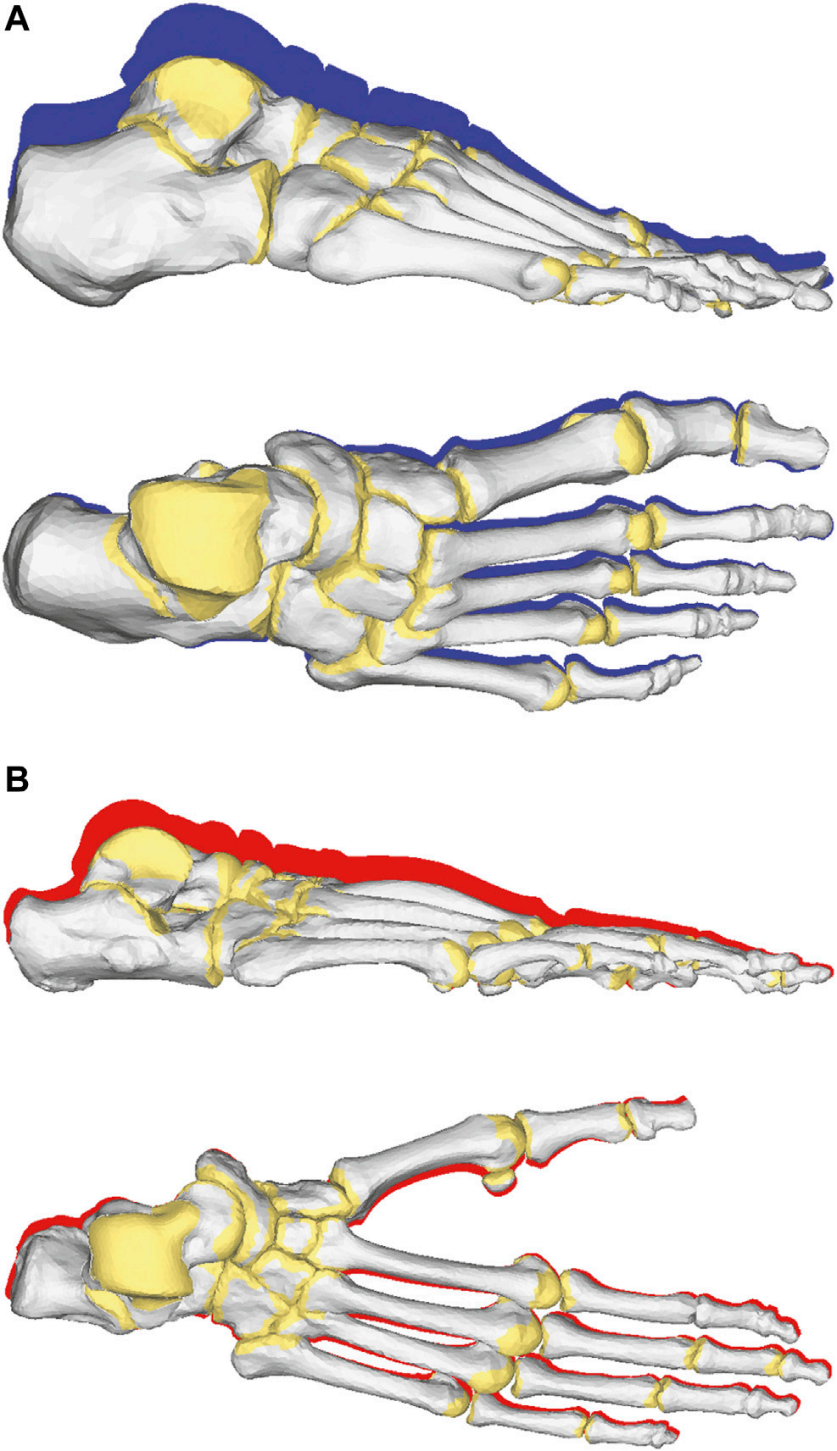
Foot bone movements from respective initial positions during quiet standing in human **(A)** and chimpanzee **(B)** feet. Red and blue shades indicate foot bone contours at initial positions.

During quiet standing, the vertical displacements of the foot bones in the inferior direction were much greater in the human foot than in the chimpanzee foot, indicating that the human foot was more deformable during axial compression (Figure 4 and Table 1). The eversions of the four human tarsal bones with respect to the global coordinate frame were generally much greater than those of the chimpanzee (Table 2). However, the plantarflexions and internal rotations of the tarsal bones were greater in the chimpanzee foot than in the human foot. In the subtalar joint, the human calcaneus everted more and rotated externally, but plantarflexed less with respect to the talus as compared with that of the chimpanzee (Table 3). A greater dorsiflexion of the calcaneocuboid joint was observed in the chimpanzee foot. In the talonavicular joint, the human navicular everted and rotated externally with respect to the talus as compared with that of the chimpanzee; this was similarly observed in the subtalar joint.

**TABLE 1:** Translational displacements of foot tarsal bones (calcaneus, talus, cuboid, and navicular) during quiet standing of human and chimpanzee; positive values indicate superior, anterior, medial directions.

		Human [mm]	Chimpanzee [mm]
Calcaneus	SUP/INF	−12.49	−6.99
	ANT/POS	−1.49	0.83
	MED/LAT	0.06	−2.18
Talus	SUP/INF	−14.63	−10.18
	ANT/POS	−1.13	2.76
	MED/LAT	1.72	0.10
Cuboid	SUP/INF	−8.73	−9.39
	ANT/POS	−0.53	0.46
	MED/LAT	−1.60	1.34
Navicular	SUP/INF	−13.85	−12.04
	ANT/POS	−0.98	0.51
	MED/LAT	0.54	0.57

**TABLE 2:** Angular displacements of foot tarsal bones (calcaneus, talus, cuboid, and navicular) during quiet standing of human and chimpanzee; positive values indicate inversion, plantarflexion, internal rotations.

		Human [°]	Chimpanzee [°]
Calcaneus	INV/EV	−2.36	0.77
	PF/DF	0.94	7.60
	IR/ER	1.29	5.24
Talus	INV/EV	2.15	1.28
	PF/DF	−0.30	3.68
	IR/ER	4.65	5.06
Cuboid	INV/EV	−9.23	−4.98
	PF/DF	−0.87	3.01
	IR/ER	−3.60	1.39
Navicular	INV/EV	−7.08	−3.76
	PF/DF	−4.50	0.79
	IR/ER	−0.20	2.62

**TABLE 3:** Angular displacements of foot tarsal joint (subtalar, calcaneocuboid, navicular joint) during quiet standing of human and chimpanzee; positive values indicate inversion, plantarflexion, and internal rotations.

		Human [°]	Chimpanzee [°]
Subtalar	INV/EV	−4.39	−0.16
	PF/DF	1.60	3.94
	IR/ER	−3.42	0.10
Calcaneocuboid	INV/EV	−6.91	−6.14
	PF/DF	−1.64	−4.05
	IR/ER	−4.97	−3.80
Talonavicular	INV/EV	−9.54	−5.27
	PF/DF	−3.46	−2.45
	IR/ER	−4.74	−2.40

The comparison of the plantar pressure distributions of human and chimpanzee feet demonstrated that, in the chimpanzee foot, the weight was primarily supported by the rear foot; hence, the plantar pressure at the heel was higher than that of the human foot (Figure 3B). Meanwhile, in the human foot, pressure was observed rather uniformly on the entire plantar surface of the foot (Figure 3A). Consequently, the COP of the human foot was located more anteriorly as compared with that of the chimpanzee.

**FIGURE 5:**
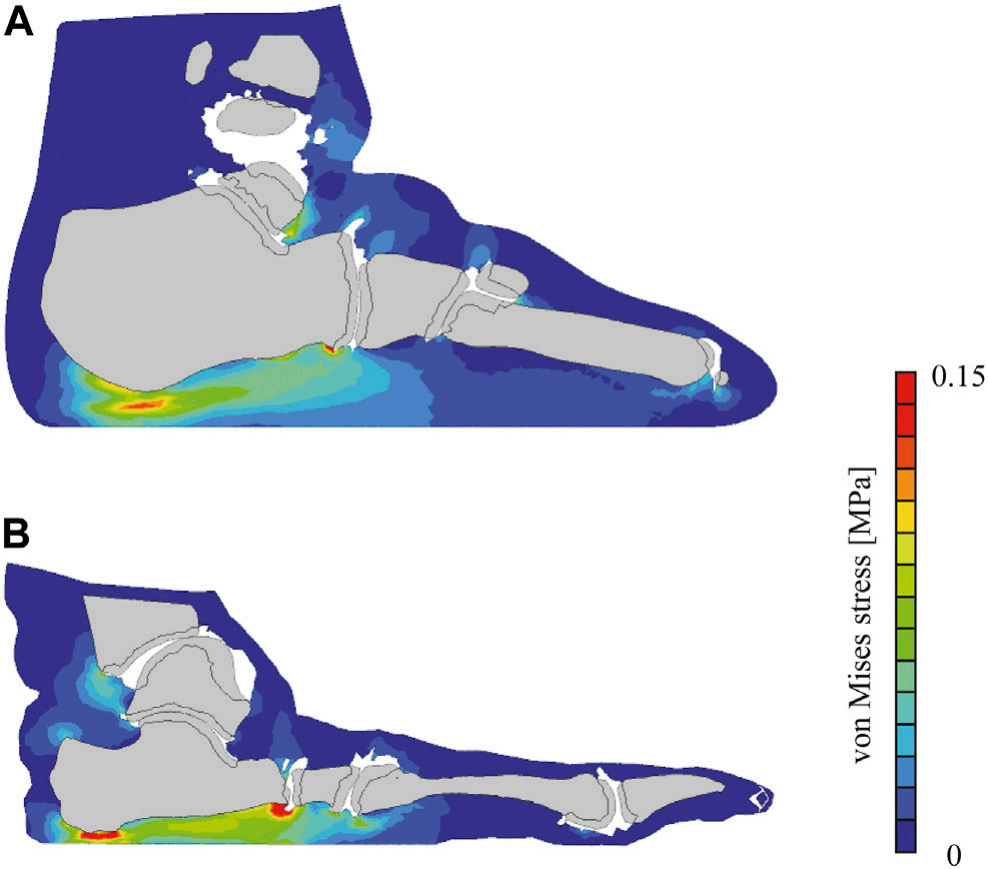
Simulated von Mises stress distribution in human **(A)** and chimpanzee **(B)** feet during simulated quiet standing. Cross section defined as plane including calcaneal tuberosity and fifth metatarsal head.

The comparison of the von Mises stress distributions of the plantar soft tissue on the vertical cross-sectional plane indicated that the calcaneal tuberosity and the fifth metatarsal head between the human and chimpanzee feet during quiet standing (Figure 5). A greater stress was generated under the calcaneus in the chimpanzee foot, indicating that a comparatively greater force was supported by the rear foot in the chimpanzee foot than in the human foot, as shown in Figure 3.

**FIGURE 6:**
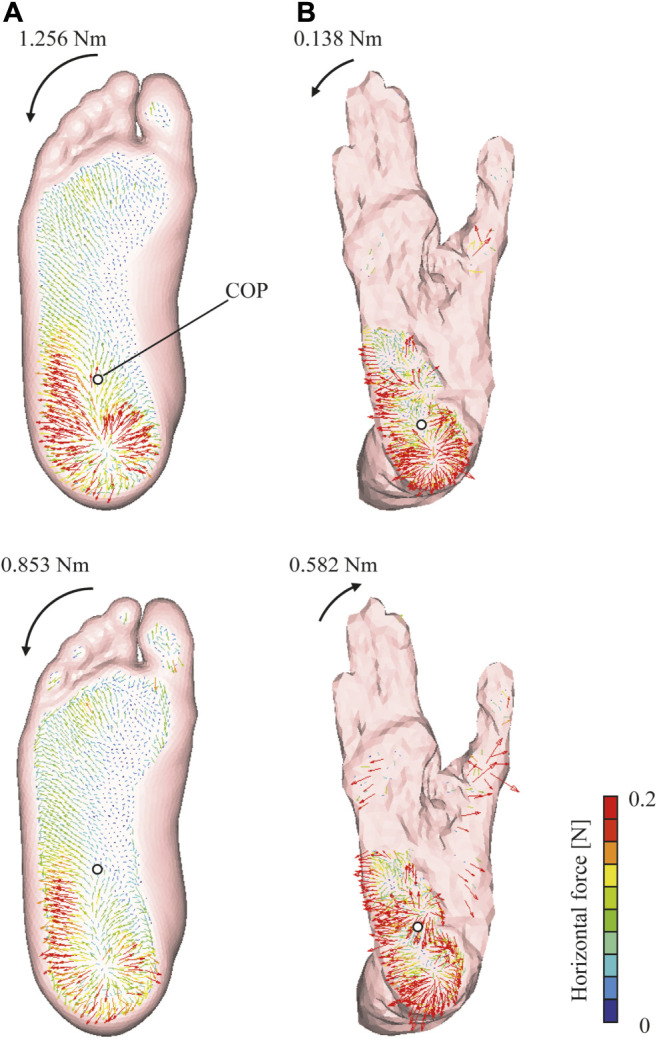
Horizontal ground reaction force vector distribution in human **(A)** and chimpanzee **(B)** feet. Upper and lower rows represent force vector distributions of axially loaded foot without tendon traction and during quiet standing, respectively. Vectors illustrate forces exerted to ground from foot. Color or length of vector represents force magnitude. Calculated COP depicted as white circle. Magnitude and direction of VFM around COP are shown.

As observed previously, greater shear forces were exerted in the rear foot region of the chimpanzee foot during quiet standing, unlike the case for the human foot (Figure 6). The VFM around the COP applied to the foot during axial loading was generated in the direction of internal rotation in both foot models; however, the magnitude was greater in the human foot than in the chimpanzee foot. The magnitudes of the VFM in the internal rotation reduced when Achilles tendon traction was present, and a change in the direction of the VFM was observed in the chimpanzee foot.

**FIGURE 7:**
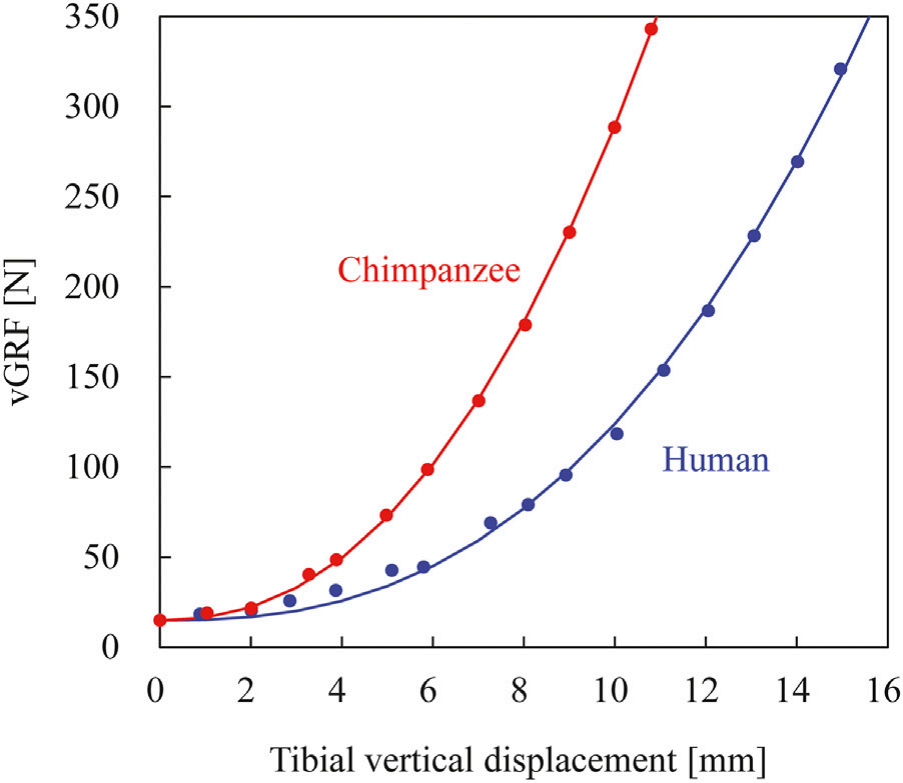
Simulated force–displacement curves of human and chimpanzee feet during axial loading without tendon traction. Blue and red lines represent those of human and chimpanzee, respectively. Curve fitted using exponential functions (see *Methods* section).

The comparison of the force–displacement curves of the human and chimpanzee feet during axial loading demonstrated that the human foot was more vertically deformable than the chimpanzee foot under the applied load (Figure 7). The elastic energy stored during axial loading (350 N) calculated based on the obtained force–displacement curves were 1.72 and 1.29 J in the human and chimpanzee feet, respectively, suggesting that the human foot was more capable of storing elastic energy during axial loading.

## Discussion

In the present study, a 3D FE model of the human and chimpanzee feet was developed to comparatively investigate the morphological adaptation of the human foot, which presumably adapted to efficient bipedal locomotion. The development of both foot models allows the prediction of foot bone movements and forces applied to the plantar surface of the foot as well as the joint contact forces under specified boundary conditions, in addition to their difference in terms of locomotor functions. Based on comparison, our simulation results and the experimental data of human cadaver specimens and a living participant during quiet standing were, if not perfectly matched, consistent with each other. Therefore, the development of the two models may provide a useful framework for understanding the functional significance and evolution of the human foot.

This study demonstrated that the transmission of the axial load from the foot to the ground was markedly different between the human and chimpanzee foot models. Specifically, the human foot was more capable of supporting the body weight by the entire plantar surface of the foot; hence, the resultant COP during quiet standing was situated more anteriorly as compared with that of the chimpanzee foot. By contrast, the body weight was supported more by the rear foot in the chimpanzee foot; consequently, the COP was located more posteriorly. This might be because the human foot exhibits a relatively high longitudinal arch, unlike the chimpanzee foot. A two-dimensional truss model of the human foot (Wright and Rennels, 1964; Sarrafian, 1987; Wang and Crompton, 2004) predicts that a greater force is applied to the anterior foot if the arch of the foot is higher, indicating that the more anterior location of the COP in the human foot is likely due to the longitudinal arch structure. If the COP is shifted anteriorly so that it is in the middle of the plantar surface, then the margin of postural stability, i.e., the distance between the COP and the edge of the plantar surface of the foot, increases. The evolution of the longitudinal arch is generally regarded as an adaptation for efficient bipedal locomotion in humans; the flattening of the arch and the elongation of the well-developed plantar aponeurosis in humans allow mechanical energy to be stored in the form of elastic energy, which is successively released during locomotion (Ker et al., 1987; Stearne et al., 2016). Additionally, the present study confirmed that the human foot was more capable of storing more elastic energy during axial loading compared with the chimpanzee foot. However, it was demonstrated that the longitudinal arch can be considered as an adaptation to achieve a more stable bipedal posture, as it anteriorly shifts the COP and increases the margin of stability. The present finding corroborates with that of Wang and Crompton (2004), who reported that the human foot proportion and presence of high arch structure are advantageous for minimizing the required muscle force during quiet standing.

Our present simulation study indicated that a VFM of 1.26 N m was exerted to the human foot model in the direction of internal rotation during axial loading. This result is consistent with previous experimental data, which indicated that the axial loading of the human cadaver lower leg generated a VFM of 1.66 N m in the direction of internal rotation when an axial load of 450 N was applied (Seki et al., 2018). Therefore, the present study supports the hypothesis that the human foot exhibits an embedded structure that allows a VFM to be generated in the direction of internal rotation. However, it was predicted that the capacity to generate a VFM was limited in the chimpanzee foot (Figure 6). The differences of the VFM between the human and chimpanzee feet were greater than 1 N m, which is substantial compared to the VFM applied to the foot during human walking (approximately 3 N m) (Collins et al., 2009). This likely indicates that the capacity to generate a VFM is unique to the human foot, which could be an adaptation to habitual bipedal locomotion (Negishi et al., 2021). During human bipedal walking and running, a VFM is generated because of trunk rotation and swing leg motion (Holden and Cavanagh, 1991; Li et al., 2001; Milner et al., 2006; Umberger, 2008; Almosnino et al., 2009). Furthermore, it has been confirmed that the magnitude of the VFM is actively controlled during human walking by arm swinging (Collins et al., 2009) and the foot orientation (Almosnino et al., 2009). Therefore, such a structurally embedded moment-generating mechanism might be an adaptation to efficient bipedal locomotion, possibly through the cancelation of the moment due to trunk rotation and swing leg motion during walking. However, the manner by which this mechanism facilitates the generation of bipedal locomotion is yet to be elucidated and should be further investigated.

The generation of the VFM may be associated with the innate mobility of the human foot during axial loading (Olerud and Rosendahl, 1987; Hintermann et al., 1994; Ito et al., 2017). The human calcaneus is known to be characterized by the plantarly-positioned lateral plantar process (Latimer and Lovejoy, 1989; Boyle et al., 2018). Therefore, the horizontal distance between the talus, where the axial load is applied, and the plantar process of the calcaneus in the coronal plane is relatively longer in the human foot than in the chimpanzee foot, causing the human calcaneus to rotate in the everting direction more easily during axial loading. Consequently, the talus rotates internally on the tilted subtalar joint surface, and so does the tibia; these coupling motions of the three bones was successfully reproduced in the simulation study (Tables 2, 3). The navicular was more externally rotated with respect to the talus in the human foot. However, in the chimpanzee foot, calcaneal eversion did not occur primarily because the calcaneal tuberosity was inverted. Therefore, the generation of the VFM in the human foot can be associated with the morphological features of the human calcaneus, including the internal rotation of the talus during axial loading.

Another finding from the present study was that the calcaneus was more plantarflexed in the chimpanzee foot than in the human foot (Table 2). Consequently, the dorsiflexion of the cuboid with respect to the calcaneus was greater in the chimpanzee foot (Table 3), indicating that the chimpanzee’s calcaneocuboid joint is mobile in the direction of plantar dorsiflexion. It has been well accepted that an excessive midfoot dorsiflexion, known as “midtarsal break,” is present during chimpanzee bipedal walking because chimpanzees have a more compliant midfoot joint for locomotion in the arboreal environment (Elftman and Manter, 1935; DeSilva, 2010; Holowka et al., 2017). By contrast, the calcaneocuboid joint of the human foot is less mobile owing to the medial prominence on the proximal joint surface of the cuboid (Bojsen-Møller, 1979). This bony feature has been recognized as one of the morphological adaptations for efficient push-off during human bipedal walking. The present results corroborate with the differences in midfoot mobility between the two species. However, the current simulation of quiet standing is not appropriate for investigating the difference in the mobility of the midfoot and the effect of the difference on the generation of propulsive forces in the late stance phase of bipedal locomotion. Hence, we will conduct further investigations by performing dynamic simulations of human and chimpanzee walking based on the foot models developed in this study.

This study demonstrated that the human foot was more capable of storing elastic energy than the chimpanzee foot during axial loading. However, the capacity for the chimpanzee foot to store elastic energy may not be so restricted during locomotion. Bennett et al. (1989) suggested that elastic energy could be stored in the foot when the midtarsal break occurs during contact with the ground because the midfoot dorsiflexion stretches the plantar ligaments and fascia longer than usual. This concept was originally demonstrated in the feet of monkeys and lesser apes (Vereecke and Aerts, 2008), but it is possible that chimpanzee feet can store more elastic energy in similar fashion. This must also be investigated by performing dynamic simulations based on the developed foot models.

The present study has some limitations. First, the CT scan data of a single individual were used to construct the foot model of each species; therefore, the variability of the foot morphology among individuals was not considered in the present study. However, since the inter-individual variability of the foot morphology within the same species should be much smaller than the inter-specific variability, the abovementioned factor should not significantly affect the present study. Second, all the material properties of the hard and soft tissues necessary for the FE analysis of the human foot were obtained from different sources, rather than being obtained from the CT-scanned participant. Therefore, size adjustment of the parameters such as ligament cross-sectional areas was necessary, but such scaling was not conducted in the present study due to the lack of information necessary for reliable scaling. In addition, these human values were used for the chimpanzee foot model because no corresponding values have been reported for chimpanzees. The use of the same parameters for the two models may have been effective for determining the effects of the morphological and structural differences between the two species on the mechanical interaction of the foot with the floor or substrate. Nevertheless, the corresponding material parameters should be obtained in future studies. Third, the modeling of the cartilage, the PA, and the encapsulated soft tissue was not completely accurate. The cartilage should be modeled as a thin cartilaginous layer of 1.0–1.5 mm thickness on the articular surfaces (Shepherd and Seedhom, 1999), but here we assigned the elements on the articular surfaces as cartilage for the sake of simplicity. In addition, although recent studies clearly indicated that the PA consists of the central and lateral bands (Guo et al., 2018; Sichting et al., 2020), only the central band was incorporated in the present model. Furthermore, the soft tissues in the foot were modeled as a homogenous material having the same material properties but the soft tissues actually consist of tissues such as fat, muscle and tendon having different material properties. The effects of such simplifications on the present findings should be confirmed in future studies. Fourth, the boundary conditions for the quiet standing simulation may not be completely accurate because muscle forces, except for the tricep surae muscle, were not considered in the present study. Although the exclusion of the abovementioned muscles might not impose a significant effect, they should be included ideally in future studies to perform more realistic analyses.

## Data Availability

The raw data supporting the conclusions of this article will be made available by the authors, without undue reservation. The dataset is available upon request from the corresponding author.
