# Supplementary material for: Comparative Functional Morphology of Human and Chimpanzee Feet Based on Three-Dimensional Finite Element Analysis
Source: Front Bioeng Biotechnol. 2022 Jan 13;9:760486. doi: 10.3389/fbioe.2021.760486 (PMC8793834; doi:10.3389/fbioe.2021.760486)
Supplement: Supplementary file 1 [file DataSheet1.PDF]

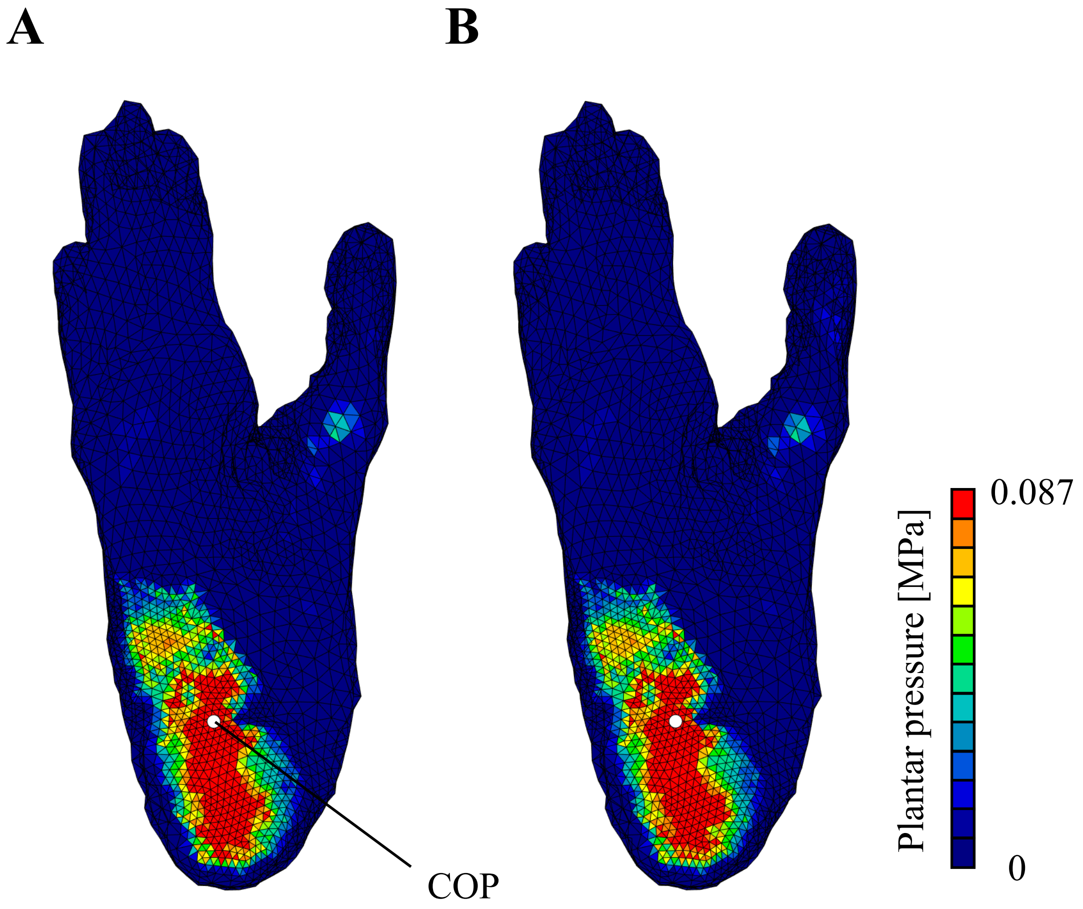

Figure S1. Plantar pressure distributions of chimpanzee feet during simulated quiet standing with the spring constants of the PA assigned to be (A) one-tenth (10%) and (B) one-half (50%) of the human values. Calculated center of pressure (COP) depicted as white circle.

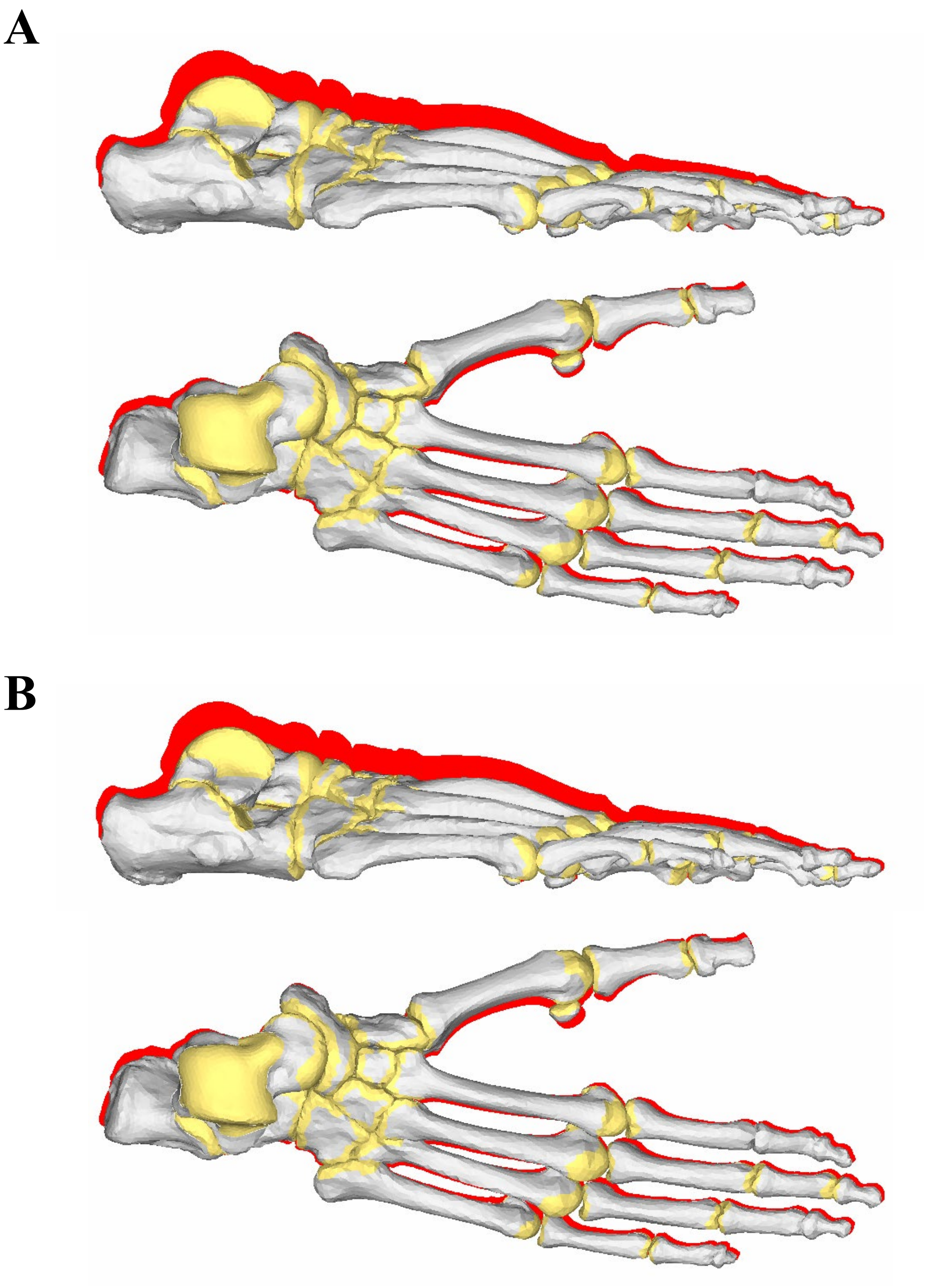

Figure S2. Foot bone movements from respective initial positions during quiet standing in chimpanzee feet with the spring constants of the PA assigned to be (A) one-tenth (10%) and (B) one-half (50%) of the human values. Red shades indicate foot bone contours at initial positions.

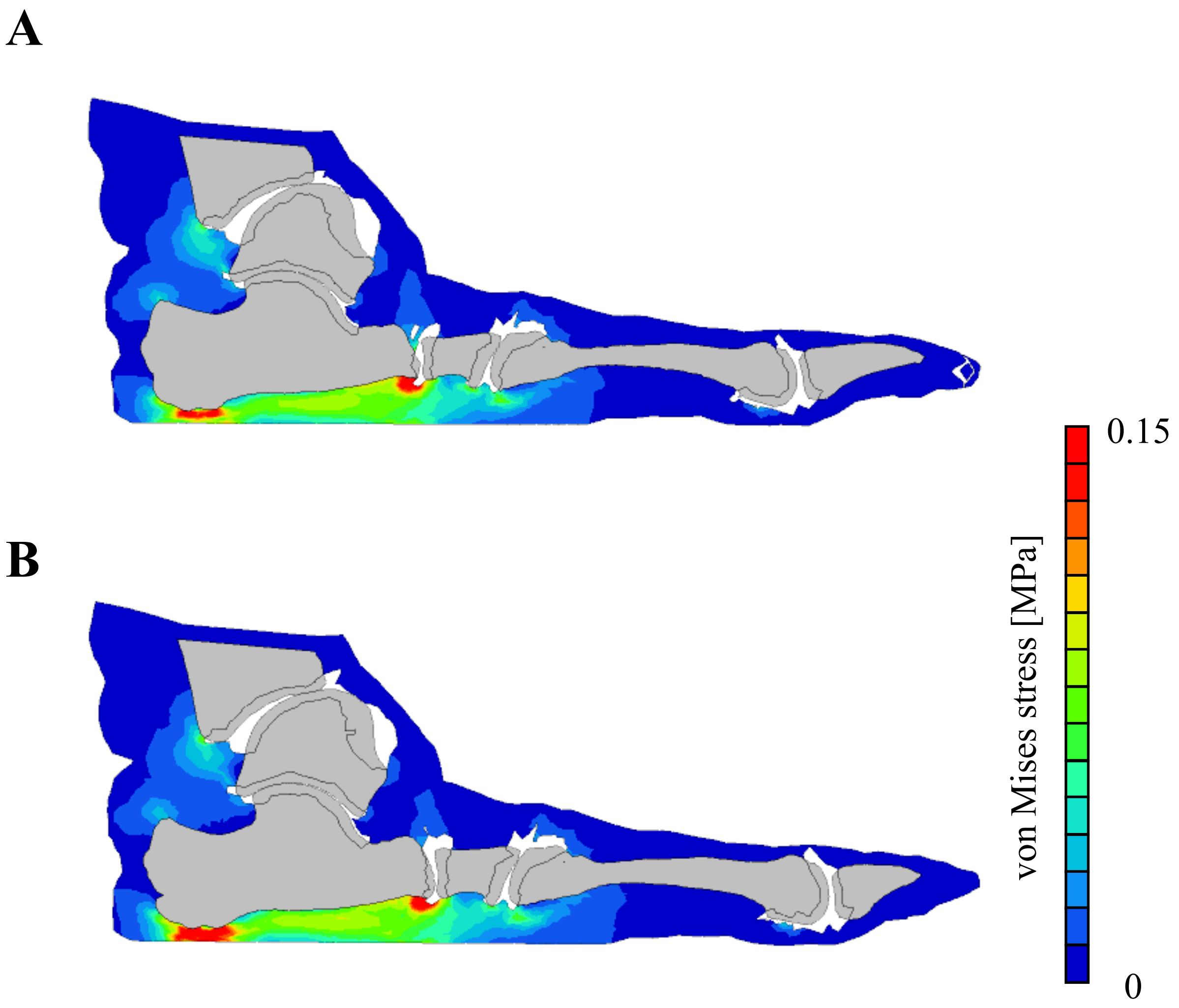

Figure S3. Simulated von Mises stress distribution in chimpanzee feet with the spring constants of the PA assigned to be (A) one-tenth (10%) and (B) one-half (50%) of the human values. Cross section defined as plane including calcaneal tuberosity and fifth metatarsal head.

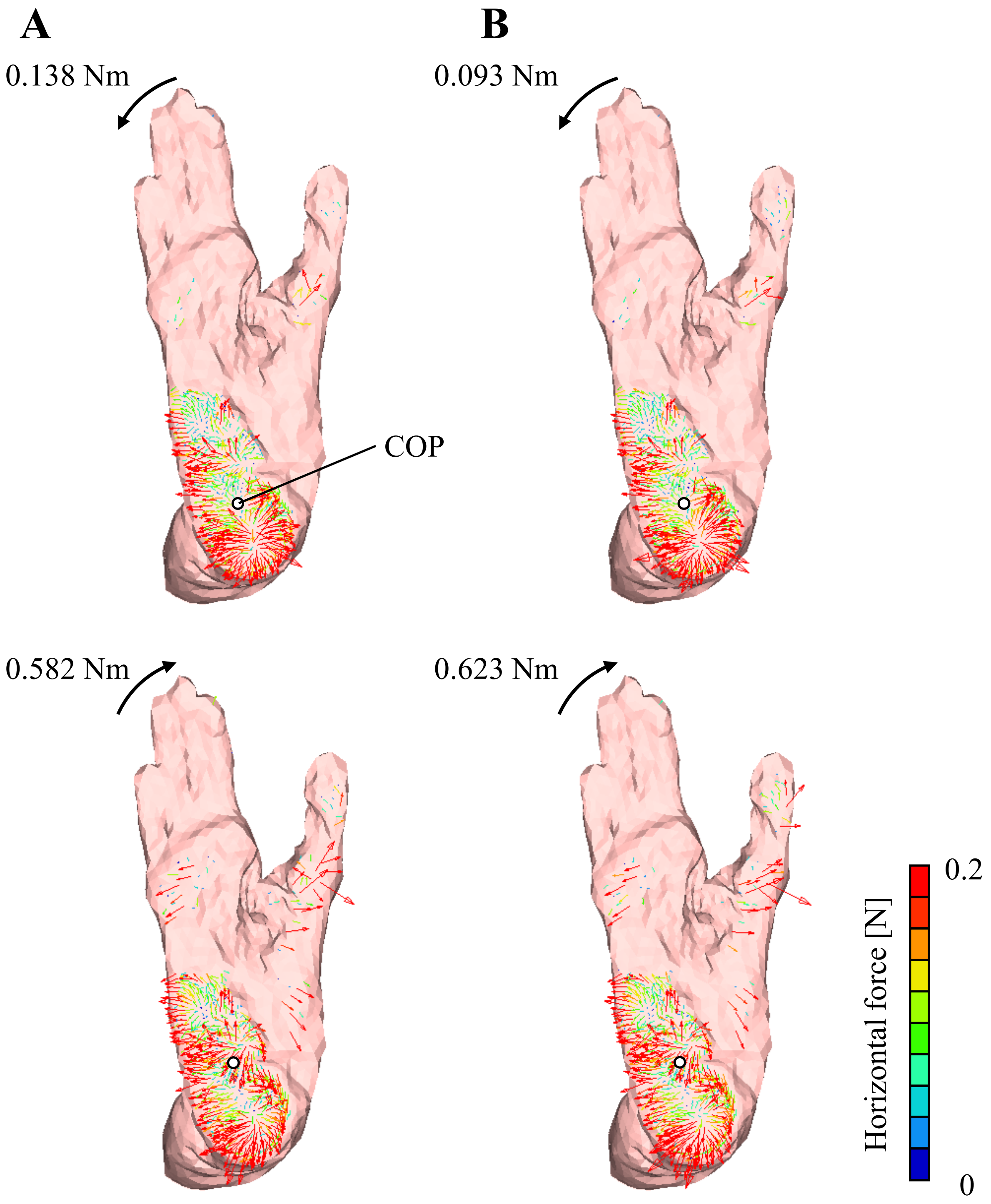

Figure S4. Horizontal ground reaction force vector distribution in chimpanzee feet with the spring constants of the PA assigned to be (A) one-tenth (10%) and (B) one-half (50%) of the human values. Upper and lower represent force vector distributions of axially loaded foot without tendon traction and during quiet standing, respectively. Vectors illustrate forces exerted to ground from foot. Color or length of vector represents force magnitude. Calculated COP depicted as white circle. Magnitude and direction of VFM around COP are shown.

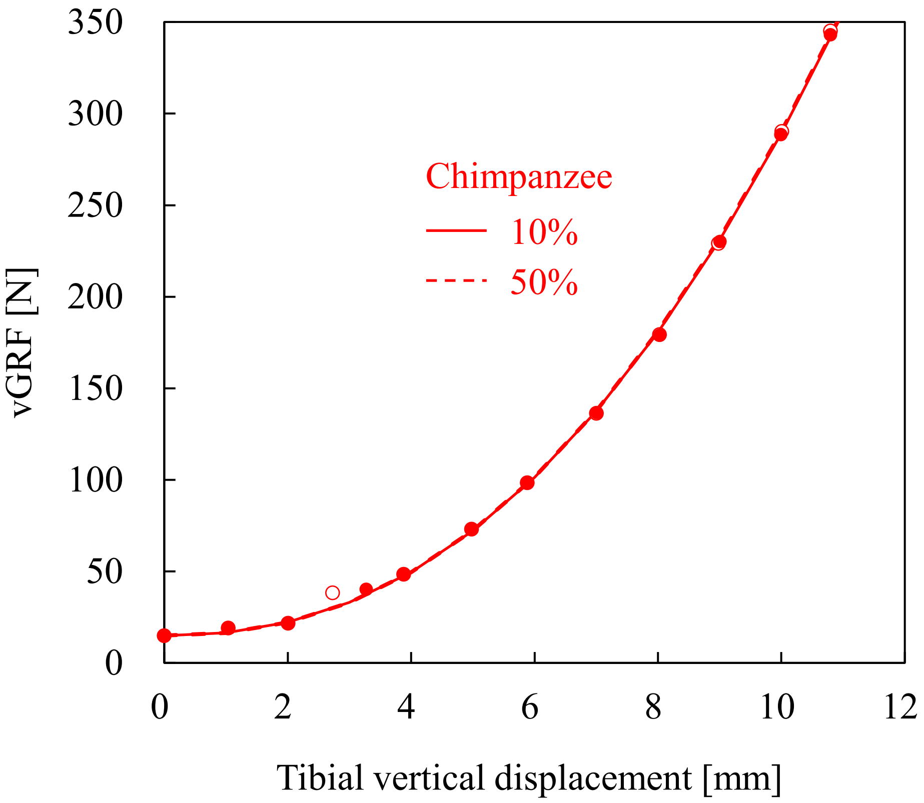

Figure S5. Simulated force-displacement curves of chimpanzee feet during axial loading without tendon traction. Solid and dashed lines represent those of chimpanzee feet with the spring constants of the PA assigned to be one-tenth (10%) and one-half (50%) of the human values. Curve fitted using exponential functions (see Methods section).

Table S1. Translational displacements of foot tarsal bones (calcaneus, talus, cuboid, and navicular) during quiet standing of chimpanzee feet with the spring constants of the PA assigned to be one-tenth (10%) and one-half (50%) of the human values; positive values indicate superior, anterior, medial directions.

		10% model [mm]	50% model [mm]
Calcaneus	SUP/INF	-6.99	-7.01
	ANT/POS	0.83	0.89
	MED/LAT	-2.18	-2.16
Talus	SUP/INF	-10.18	-10.13
	ANT/POS	2.76	2.73
	MED/LAT	0.10	0.05
Cuboid	SUP/INF	-9.39	-9.26
	ANT/POS	0.46	0.48
	MED/LAT	1.34	1.12
Navicular	SUP/INF	-12.04	-11.84
	ANT/POS	0.51	0.56
	MED/LAT	0.57	0.47

Table S2. Angular displacements of foot tarsal bones (calcaneus, talus, cuboid, and navicular) during quiet standing of chimpanzee feet with the spring constants of the PA assigned to be one-tenth (10%) and one-half (50%) of the human values; positive values indicate inversion, plantarflexion, internal rotations.

		10% model [°]	50% model [°]
Calcaneus	INV/EV	0.77	0.90
	PF/DF	7.60	7.41
	IR/ER	5.24	5.00
Talus	INV/EV	1.28	1.31
	PF/DF	3.68	3.36
	IR/ER	5.06	4.80
Cuboid	INV/EV	-4.98	-4.97
	PF/DF	3.01	3.02
	IR/ER	1.39	1.04
Navicular	INV/EV	-3.76	-3.58
	PF/DF	0.79	0.67
	IR/ER	2.62	2.43

Table S3. Angular displacements of foot tarsal joint (subtalar, calcaneocuboid, navicular joint) during quiet standing of chimpanzee feet with the spring constants of the PA assigned to be one-tenth (10%) and one-half (50%) of the human values; positive values indicate inversion, plantarflexion, and internal rotations.

		10% model [°]	50% model [°]
Subtalar	INV/EV	-0.16	-0.06
	PF/DF	3.94	4.06
	IR/ER	0.10	0.11
Calcaneocuboid	INV/EV	-6.14	-6.22
	PF/DF	-4.05	-3.86
	IR/ER	-3.80	-3.90
Talonavicular	INV/EV	-5.27	-5.10
	PF/DF	-2.45	-2.28
	IR/ER	-2.40	-2.33
